# Lipopolysaccharide-binding protein is associated with arterial stiffness in patients with type 2 diabetes: a cross-sectional study

**DOI:** 10.1186/s12933-017-0545-3

**Published:** 2017-05-10

**Authors:** Takeshi Sakura, Tomoaki Morioka, Atsushi Shioi, Yoshinori Kakutani, Yuya Miki, Yuko Yamazaki, Koka Motoyama, Katsuhito Mori, Shinya Fukumoto, Tetsuo Shoji, Masanori Emoto, Masaaki Inaba

**Affiliations:** 1 Department of Metabolism, Endocrinology and Molecular Medicine, Osaka City University Graduate School of Medicine, 1-4-3, Asahi-machi, Abeno-ku, Osaka, 545-8585 Japan; 2 Department of Vascular Medicine, Osaka City University Graduate School of Medicine, 1-4-3, Asahi-machi, Abeno-ku, Osaka, 545-8585 Japan; 3 Vascular Science Center for Translational Research, Osaka City University Graduate School of Medicine, 1-4-3, Asahi-machi, Abeno-ku, Osaka, 545-8585 Japan; 4 Department of Nephrology, Osaka City University Graduate School of Medicine, 1-4-3, Asahi-machi, Abeno-ku, Osaka, 545-8585 Japan; 5 Department of Premier Preventive Medicine, Osaka City University Graduate School of Medicine, 1-4-3, Asahi-machi, Abeno-ku, Osaka, 545-8585 Japan

**Keywords:** Lipopolysaccharide-binding protein, Arterial stiffness, Pulse wave velocity, Type 2 diabetes

## Abstract

**Background:**

Lipopolysaccharide (LPS)-binding protein (LBP) is an acute-phase reactant that mediates immune responses triggered by LPS. Recent evidence indicates the association of circulating LBP levels with obesity, diabetes, and cardiovascular diseases. In this study, we aimed to investigate the relationship between serum LBP levels and arterial stiffness in patients with type 2 diabetes.

**Methods:**

A total of 196 patients with type 2 diabetes, including 101 men and 95 women, were enrolled in this cross-sectional study. Fasting serum LBP levels were determined by enzyme-linked immunosorbent assay. Arterial stiffness was assessed by measuring the aortic pulse wave velocity (PWV).

**Results:**

The mean values of serum LBP and aortic PWV were 18.2 μg/mL and 1194 cm/s, respectively. Serum LBP levels were positively correlated with body mass index, triglycerides, high-sensitivity C-reactive protein, and insulin resistance index and were negatively correlated with high-density lipoprotein cholesterol. They were, however, not significantly correlated with aortic PWV in univariate analyses. Multivariate analysis revealed that serum LBP levels were independently and positively associated with aortic PWV (*β* = 0.135, *p* = 0.026) after adjusting for age, sex, body mass index, albumin, high-sensitivity C-reactive protein, and other cardiovascular risk factors. Further analyses revealed that the impact of serum LBP levels on aortic PWV was modified by sex, and the association between serum LBP levels and aortic PWV was found to be significant only in men.

**Conclusions:**

Serum LBP levels are associated with arterial stiffness, independent of obesity and traditional cardiovascular risk factors, especially in men with type 2 diabetes. This study indicates a potential role of the LPS/LBP-induced innate immunity in the development and progression of arterial stiffness in type 2 diabetes.

## Background

The association of chronic inflammation with the pathogeneses of obesity, diabetes, and atherosclerosis is well recognized [1]. Accumulating evidence indicates a link between low-grade inflammation produced by common subclinical or chronic infections and the risk of atherosclerosis in humans [2, 3]. Lipopolysaccharide (LPS) derived from Gram-negative bacteria is known to play a critical role in triggering the immune and inflammatory responses in vascular cells, leading to atherosclerosis [4]. LPS-binding protein (LBP), an acute-phase reactant synthesized mainly in the liver, binds LPS and initiates the immune response by presenting LPS to cluster of differentiation (CD)14, which in turn interacts with toll-like receptor (TLR)4 on immune cells [5]. Since LBP is synthesized and released into circulation in the presence of LPS, with a relatively long half-life, LBP level is considered a surrogate biomarker for the activation of LPS-induced innate immune responses [6–8].

Several studies in humans recently demonstrated that serum LBP levels are closely associated with obesity, the metabolic syndrome, and type 2 diabetes [6, 8–13]. Furthermore, serum LBP levels were shown to be a predictor of prevalent coronary artery disease [7] and cardiovascular mortality [14], independent of established cardiovascular risk factors and markers of systemic inflammation. A recent study also showed that serum LBP levels were positively correlated with subclinical atherosclerosis, as assessed by carotid intima-media thickness, in healthy individuals, independent of body mass index (BMI) and high-sensitivity C-reactive protein (hs-CRP) level [15]. These studies collectively indicate that serum LBP level may be a biomarker for atherosclerotic cardiovascular disease and implicate a potential role of the innate immune mechanisms in the progression of atherosclerosis in humans.

To our knowledge, no previous study has investigated the relationship between LBP and arterial stiffness, a well-established surrogate marker for cardiovascular diseases [16], in human subjects. Moreover, no study has examined the impact of LBP on subclinical atherosclerosis in patients with comorbid cardiovascular risk factors, including diabetes. Therefore, in the present study, we investigated the association between serum LBP levels and arterial stiffness by measuring aortic pulse wave velocity (PWV) in patients with type 2 diabetes.

## Methods

### Study design and subjects

We consecutively enrolled 196 patients with type 2 diabetes, including 101 men and 95 women, who were admitted to the Diabetes Center of the Osaka City University Hospital for the purpose of glycemic control, education, and/or evaluation of diabetic complications between July 2013 and December 2015. Type 2 diabetes was diagnosed based on American Diabetes Association criteria [17]. Patients with type 1 diabetes or other types of diabetes were not included in the present study. The following patients were not included in this study: those with acute or chronic infection, chronic inflammatory disease, use of anti-inflammatory drugs including glucocorticoids, or hematologic or malignant disease, and those who underwent recent surgery within 1 month at the time of entry. A smoker was defined as a current smoker in our analyses.

### Physical and laboratory measurements

Blood pressure was determined using an automatic sphygmomanometer with a conventional cuff after the subject had rested for at least 5 min. Waist circumference was measured to the nearest centimeter at the level of the umbilicus in a standing position at the end of gentle expiration. Blood was drawn after an overnight fast and biochemical parameters were analyzed using a standard laboratory method at the Central Clinical Laboratory of the Osaka City University Hospital [18, 19]. The estimated glomerular filtration rate (eGFR) was calculated using the Japanese eGFR equation [20]. Immunoreactive insulin levels were measured for subjects not receiving insulin therapy (n = 144) by electro-chemiluminescence immunoassay [Cobas 8000 (502/602), Roche Diagnostics] at the Central Clinical Laboratory. Homeostasis model assessment of insulin resistance (HOMA-R) was calculated according to the following formula: fasting insulin (μU/mL) × fasting glucose (mg/dL)/405 [21]. Frozen serum samples were shipped to SRL Inc. (Tokyo, Japan) and hs-CRP concentrations were measured by means of particle-enhanced immunonephelometry with the Behring nephelometer using N Latex CRP mono reagent.

Serum levels of LBP were measured using a commercial enzyme-linked immunosorbent assay (HK315-02, HyCult Biotech Inc., Uden, the Netherlands) as per manufacturer’s instructions. The intra- and inter-assay coefficients of LBP variation were <5 and <10%, respectively.

### Measurement of arterial stiffness

Arterial stiffness was evaluated by measuring PWV in the heart-femoral segment using an automatic waveform analyzer (Model BP-203RPE; Omron Colin Co., Ltd., Tokyo, Japan) as described previously [22, 23]. Reproducibility in the measurements of arterial stiffness was confirmed in our previous study, in which the coefficients of variation were less than 5% for heart-femoral PWV [22].

### Statistical analysis

Data are expressed as the number (%), mean ± standard deviation (SD), or median (interquartile range) as appropriate. For comparisons between men and women, χ^2^-test, unpaired *t*-test, or Wilcoxon rank-sum test, was performed as appropriate. Skewed parameters, such as HOMA-R, triglycerides, and hs-CRP were logarithmically transformed before regression analysis. Simple linear regression analyses were performed to evaluate the relationship between serum LBP levels or aortic PWV and various clinical variables, including cardiovascular risk factors. To explore the association between LBP and aortic PWV, multiple linear regression analyses were performed after adjustment for age, sex, BMI, systolic blood pressure, albumin, eGFR, high-density lipoprotein (HDL) cholesterol level, log [hs-CRP], serum LBP level, treatment with statins, treatment with inhibitors of the renin-angiotensin system (RAS inhibitors), treatment with calcium-channel blockers, and smoking status. To assess whether the effect of serum LBP levels on aortic PWV is modified by sex, the interaction term between LBP and sex was inserted into the multiple regression model. A *p* value of <0.20 was considered significant for interaction effects, as has been used in a previous study [24], and a *p* value of <0.05 was considered significant for all other analyses. Statistical analyses were performed by using the JMP 10 software program (SAS Institute Inc., Cary, NC, USA).

## Results

### Clinical characteristics, serum LBP levels, and aortic PWV of the subjects

The clinical characteristics of the total population, as well as of men and women, are shown in Table 1. The subjects had a mean age of 61 years, median duration of diabetes of 10 years, and mean BMI of 27.1 kg/m^2^. One hundred sixty-eight subjects (85.7%) were receiving any antihyperglycemic agents. Eighty (40.8%) subjects were treated with statins for dyslipidemia, 72 (36.7%) with RAS inhibitors, and 74 (37.8%) with calcium-channel blockers for hypertension. There were significantly more male smokers than female smokers. Serum creatinine levels, but not eGFR, were significantly different between men and women. Parameters of obesity and insulin resistance, such as BMI, waist circumference, and HOMA-R, were not significantly different between men and women. Triglycerides levels and diastolic blood pressure were higher, and HDL-cholesterol levels were lower in men than in women.

**Table 1:** Clinical characteristics, serum LBP levels, and aortic PWV in all subjects as well as in men and women with type 2 diabetes

	All subjects	Men	Women	*p*
*N*	196	101	95	–
Age (years)	61 ± 14	60 ± 13	62 ± 15	0.346
Duration of diabetes (years)	10 (3–18)	10 (2–20)	10 (5–16)	0.589
BMI (kg/m^2^)	27.1 ± 6.1	26.9 ± 5.5	27.3 ± 6.7	0.640
Waist circumference (cm)	92 ± 15	93 ± 13	91 ± 16	0.252
Systolic blood pressure (mmHg)	135 ± 20	135 ± 19	135 ± 20	0.962
Diastolic blood pressure (mmHg)	80 ± 12	81 ± 11	77 ± 13	0.022
Smoker *n* (%)	41 (20.9)	34 (33.7)	7 (7.4)	<0.001
Antihyperglycemic agents *n* (%)	168 (85.7)	86 (85.2)	82 (86.3)	0.816
Statins *n* (%)	80 (40.8)	38 (37.6)	42 (44.2)	0.348
RAS inhibitors *n* (%)	72 (36.7)	34 (33.7)	38 (40.0)	0.358
Calcium-channel blockers *n* (%)	74 (37.8)	35 (34.7)	39 (41.1)	0.356
Albumin (g/dL)	4.0 ± 0.5	4.1 ± 0.6	4.0 ± 0.4	0.205
Creatinine (mg/dL)	0.95 ± 0.58	1.07 ± 0.59	0.82 ± 0.55	0.002
eGFR (mL/min/1.73 m^2^)	67.3 ± 23.7	66.3 ± 21.8	68.3 ± 25.7	0.548
Fasting glucose (mg/dL)	129 ± 37	127 ± 39	132 ± 34	0.367
HbA1c (%)	8.3 ± 1.9	8.3 ± 2.0	8.3 ± 1.7	0.950
HOMA-R^a^	2.3 (1.5–3.7)	2.2 (1.5–3.4)	2.6 (1.6–3.9)	0.287
Triglycerides (mg/dL)	120 (85–163)	131 (88–176)	106 (76–156)	0.020
HDL-cholesterol (mg/dL)	45 ± 12	42 ± 10	48 ± 14	<0.001
LDL-cholesterol (mg/dL)	116 ± 42	120 ± 49	111 ± 32	0.105
hs-CRP (mg/L)	0.70 (0.27–1.58)	0.65 (0.25–1.12)	0.79 (0.29–1.95)	0.221
LBP (μg/mL)	18.2 ± 6.3	17.7 ± 5.8	18.7 ± 6.7	0.267
Aortic PWV (cm/s)	1194 ± 346	1224 ± 350	1162 ± 340	0.209

Values are expressed as *n* (%), mean ± SD, or median (interquartile range). *p* values were determined by using χ^2^-test, unpaired *t*-test, or Wilcoxon rank-sum test, as appropriate, for comparison between men and women

*LBP* lipopolysaccharide-binding protein, *PWV* pulse wave velocity, *BMI* body mass index, *RAS* renin-angiotensin system, *eGFR* estimated glomerular filtration rate, *HbA1c* glycated hemoglobin A1c, *HOMA-R* homeostasis model assessment of insulin resistance, *HDL* high-density lipoprotein, *LDL* low-density lipoprotein, *hs-CRP* high-sensitivity C-reactive protein

^a^ *N* = 144 for all subjects, *n* = 79 for men, and n = 65 for women not receiving insulin therapy

Mean ± SD value for serum LBP levels of all subjects was 18.2 ± 6.3 μg/mL (range 2.1–36.2 μg/mL). Mean ± SD value for the aortic PWV was 1194 ± 346 cm/s (range 610–2500 cm/s). Serum LBP levels and aortic PWV were not significantly different between men and women.

### Association between serum LBP levels and cardiovascular risk factors

We first examined the association of serum LBP levels with the parameters related to obesity, insulin resistance, and other cardiovascular risk factors by simple linear regression analyses (Table 2). Serum LBP levels were significantly correlated with measures of obesity including BMI (*r* = 0.279, *p* < 0.001) and waist circumference (*r* = 0.295, *p* < 0.001) and with parameters related to insulin resistance including HOMA-R (*r* = 0.257, *p* = 0.002), triglycerides (*r* = 0.234, *p* < 0.001), and HDL-cholesterol levels (*r* = −0.163, *p* = 0.020). In addition, serum LBP levels were correlated with inflammatory factors such as hs-CRP (*r* = 0.575, *p* < 0.001) and serum albumin levels (*r* = −0.156, *p* = 0.029) (Table 2).

**Table 2:** Correlation between serum LBP levels or aortic PWV and cardiovascular risk factors

	Serum LBP levels		Aortic PWV	
	*r*	*p*	*r*	*p*
Age	−0.126	0.079	0.568	<0.001
BMI	0.279	<0.001	−0.312	<0.001
Waist circumference	0.295	<0.001	−0.216	0.003
Systolic blood pressure	0.082	0.252	0.498	<0.001
Albumin	−0.156	0.029	−0.210	0.003
eGFR	−0.015	0.838	−0.473	<0.001
HbA1c	0.058	0.417	−0.034	0.634
Log [HOMA-R]^a^	0.257	0.002	−0.279	<0.001
Log [triglycerides]	0.234	<0.001	−0.108	0.131
HDL-cholesterol	−0.163	0.020	−0.021	0.769
LDL-cholesterol	0.077	0.284	−0.104	0.148
Log [hs-CRP]	0.575	<0.001	−0.053	0.462
LBP	–	–	0.065	0.364

*r* correlation coefficient determined by simple regression analysis, *LBP* lipopolysaccharide-binding protein, *PWV* pulse wave velocity, *BMI* body mass index, *eGFR* estimated glomerular filtration rate, *HbA1c* glycated hemoglobin A1c, *HOMA-R* homeostasis model assessment of insulin resistance, *HDL* high-density lipoprotein, *LDL* low-density lipoprotein, *hs-CRP* high-sensitivity C-reactive protein, *PWV* pulse wave velocity

^a^Subjects excluding those receiving insulin therapy (n = 144)

### Association of aortic PWV with cardiovascular risk factors and serum LBP levels

Next, we examined the association of aortic PWV with cardiovascular risk factors and serum LBP levels by simple linear regression analyses (Table 2). Aortic PWV was well correlated with age (*r* = 0.568, *p* < 0.001), systolic blood pressure (*r* = 0.498, *p* < 0.001), and eGFR (*r* = −0.473, *p* < 0.001). In addition, aortic PWV was found to be negatively correlated with obesity-related parameters, such as BMI (*r* = −0.312, *p* < 0.001), waist circumference (*r* = −0.216, *p* = 0.003), and HOMA-R (*r* = −0.279, *p* < 0.001). Among inflammation-related parameters, serum albumin levels were negatively correlated (*r* = −0.210, *p* = 0.003), while neither hs-CRP (*r* = −0.053, *p* = 0.462) nor serum LBP levels (*r* = 0.065, *p* = 0.364) were significantly correlated with aortic PWV in univariate analyses (Table 2).

### Multivariate analyses of the factors associated with aortic PWV

To explore whether serum LBP levels have an independent association with arterial stiffness, we performed multiple regression analyses after adjusting for age; sex; BMI; systolic blood pressure; albumin; eGFR; log [triglycerides]; HDL-cholesterol; log [hs-CRP]; use of statins; use of RAS inhibitors; use of calcium-channel blockers; and smoking status (Model 1, Table 3). Aside from age (*β* = 0.344, *p* < 0.001), BMI (*β* = −0.233, *p* = 0.001), systolic blood pressure (*β* = 0.382, *p* < 0.001), and use of RAS inhibitors (*β* = −0.130, *p* = 0.025), serum LBP levels were found to be independently and positively associated with aortic PWV (*β* = 0.135, *p* = 0.026). On the other hand, hs-CRP levels were not found to be an independent determinant of aortic PWV (*β* = 0.024, *p* = 0.733) (Model 1, Table 3). Unlike RAS inhibitors, use of calcium-channel blockers was not significantly associated with aortic PWV (*β* = 0.049, *p* = 0.412). The association between serum LBP levels and aortic PWV remained nearly significant (*β* = 0.125, *p* = 0.083) after further adjustment for log [HOMA-R] (Model 2, Table 3).

**Table 3:** Multiple regression analysis of the determinants for aortic PWV

	Model 1		Model 2	
	*β*	*p*	*β*	*p*
Age (years)	0.344	<0.001	0.327	<0.001
Sex (male = 1, female = 0)	−0.117	0.033	−0.158	0.012
BMI (kg/m^2^)	−0.233	0.001	−0.228	0.007
Systolic blood pressure (mmHg)	0.382	<0.001	0.310	<0.001
Albumin (g/dL)	−0.074	0.185	−0.132	0.051
eGFR (mL/min/1.73 m^2^)	−0.113	0.095	−0.150	0.048
Log [HOMA-R]^a^	–	–	−0.030	0.692
Log [triglycerides (mg/dL)]	−0.092	0.119	−0.105	0.151
HDL-cholesterol (mg/dL)	−0.082	0.180	−0.120	0.086
Statins (yes = 1, no = 0)	0.060	0.249	0.101	0.079
RAS inhibitors (yes = 1, no = 0)	−0.130	0.025	0.180	0.006
Calcium-channel blockers (yes = 1, no = 0)	0.049	0.412	0.056	0.396
Smokers (yes = 1, no = 0)	−0.021	0.698	−0.033	0.606
Log [hs-CRP (mg/L)]	0.024	0.733	0.005	0.950
LBP (μg/mL)	0.135	0.026	0.125	0.083
*R* ^*2*^	0.585	<0.001	0.631	<0.001

*β* standard regression coefficient determined by multiple regression analysis, *R* ^*2*^ coefficient of determination, *PWV* pulse wave velocity, *BMI* body mass index, *eGFR* estimated glomerular filtration rate, *HOMA-R* homeostasis model assessment of insulin resistance, *HDL* high-density lipoprotein, *RAS* renin-angiotensin system, *hs-CRP* high-sensitivity C-reactive protein, *LBP* lipopolysaccharide-binding protein

^a^Subjects excluding those receiving insulin therapy (n = 144)

### Separate correlations between serum LBP levels and aortic PWV in men and women

Additionally, we performed an interaction analysis to assess whether sex modified the relationship between serum LBP levels and aortic PWV. The analysis indicated a potential effect modification by sex on the association between serum LBP levels and aortic PWV (*β* = −0.137, *p* for interaction = 0.065). Then, we examined the association between serum LBP levels and aortic PWV in men (n = 101) and women (n = 95) separately. Serum LBP levels were found to be positively correlated with aortic PWV in men (*r* = 0.242, *p* = 0.015), and the correlation remained significant (*β* = 0.209, *p* = 0.011) after adjusting for age; BMI; systolic blood pressure; albumin; eGFR; log [triglycerides]; HDL-cholesterol; log [hs-CRP]; use of statins; use of RAS inhibitors; use of calcium-channel blockers, and smoking status. On the contrary, no significant correlation was found between serum LBP levels and aortic PWV in women (*β* = 0.028, *p* = 0.768). Although not statistically significant, the impact of serum LBP levels on aortic PWV was greater in men (*β* = 0.146, *p* = 0.140) than in women (*β* = −0.020, *p* = 0.874), after further adjustment for log [HOMA-R].

## Discussion

The present study demonstrated that serum LBP levels are positively associated with arterial stiffness, as assessed by aortic PWV, in patients with type 2 diabetes. Serum LBP levels were positively correlated with the parameters of obesity, insulin resistance, and inflammation in our diabetic subjects, which is in agreement with observations from previous studies of non-diabetic populations [9, 12, 15]. However, it is noteworthy that the association between serum LBP levels and aortic PWV was independent of obesity, inflammation, and other traditional cardiovascular risk factors. The results further revealed that the association between serum LBP levels and aortic PWV was observed in men, but not in women. To our knowledge, this is the first report to demonstrate the clinical implications of circulating LBP in the increased arterial stiffness in type 2 diabetes.

### Clinical association between serum LBP levels and arterial stiffness

This study clearly demonstrated that serum LBP levels are independently and positively associated with aortic PWV in patients with type 2 diabetes. Two previous studies showed that serum LBP levels were a significant predictor of prevalent coronary artery disease [7] and cardiovascular mortality [14], independent of established cardiovascular risk factors and inflammatory markers, in hospital-based cohort studies. Recently, serum LBP levels have been shown to be independently associated with carotid intima-media thickness, the most established morphological surrogate marker for cardiovascular morbidity and mortality [25, 26], after adjusting for age, sex, BMI, and hs-CRP levels, in healthy subjects [15]. Aortic PWV has also been established as an independent predictor of future cardiovascular events and mortality [27, 28]. Recent studies have shown a close association between blood pressure and arterial stiffness as evaluated by brachial-ankle PWV [29] in patients with type 2 diabetes, and a predictive value of brachial-ankle PWV on the progression of coronary artery calcification [30] The results from the present study are in agreement with those of the previous study [15] and indicate a possible role of LBP in the progression of atherosclerosis. Furthermore, this study is the first to report the impact of LBP on arterial stiffness in patients with type 2 diabetes, who have advanced arterial stiffening [22, 27, 31–33] and elevated cardiovascular mortality [34].

### Potential mechanisms underlying the relationship between LBP and arterial stiffness

Importantly, in this study, the relationship between serum LBP levels and aortic PWV was independent of traditional cardiovascular risk factors including age, obesity, renal dysfunction, hyperglycemia, and dyslipidemia. It is well recognized that low-grade inflammation is among the major factors involved in arterial stiffness in the general population [35] and in patients with type 2 diabetes [33, 36]. Recent evidence indicates that bacterial endotoxins, or LPS, are an important source of vascular inflammation in atherosclerosis [4]. In vitro studies have demonstrated that LPS induced expression of matrix metalloproteinase-9 through the TLR4/nuclear factor-κB pathway [37, 38] and stimulated the release of proinflammatory cytokines [39] in vascular smooth muscle cells [37, 39] and endothelial cells [38]. In vivo studies also showed that the blockade of LPS signaling by TLR4 antagonists reduced the infiltration of monocytes/macrophages and expression of interleukin-6 and matrix metalloproteinase-9 in the atherosclerotic lesions of diabetic mice [40]. In humans, prospective population-based studies showed that chronic infection [2] or endotoxemia [3] is a strong risk factor of carotid atherosclerosis [2, 3] and cardiovascular diseases [3]. In a large population-based study, soluble CD14, a mediator for the activation of immune cells by LPS, was independently associated with aortic PWV, but not with carotid intima-media thickness [41]. In light of the combined experimental and clinical evidence, and considering the fact that circulating LBP binds to LPS and promotes the innate immune response [5], our data may indicate a critical role of the LPS/LBP-induced inflammation in the pathogenesis of arterial stiffness in type 2 diabetes.

Alternatively, the association between serum LBP levels and aortic PWV can be explained by the indirect effects of LBP on aortic PWV via obesity and insulin resistance. Recently, moderately increased LPS in circulation, or metabolic endotoxemia, in response to a high-fat diet was shown to trigger obesity and insulin resistance in mice [42, 43]. Several studies also showed that LBP is produced by adipocytes and plays an essential role in inflammation- and obesity-associated adipose tissue dysfunction [44, 45]. The relationship between LBP and obesity, insulin resistance, and the metabolic syndrome has been demonstrated in a number of studies performed in humans in both cross-sectional [8, 9, 11, 12] and prospective [10, 13] designs. In agreement with these studies, we found that serum LBP levels were positively correlated with parameters of obesity, insulin resistance, and components of the metabolic syndrome in patients with type 2 diabetes (Table 2). Considering the evidence that the metabolic syndrome [46] and insulin resistance [33] are closely associated with increased arterial stiffness, it is conceivable that LBP affected aortic stiffness through insulin resistance in our subjects. However, in our data, the association of obesity and dyslipidemia with aortic PWV was less significant compared with that of age, hypertension, renal dysfunction, and serum LBP levels (Table 3). Moreover, HOMA-R was not independently associated with aortic PWV and an additional adjustment for log [HOMA-R] did not virtually affect the relationship between LBP and aortic PWV in the multivariate model (Model 2, Table 3). Thus, although LPS/LBP-induced immune response is commonly involved in arterial stiffness [41], obesity, and insulin resistance [6, 8–13], our results indicate that the LPS/LBP-induced innate immunity independently affects arterial stiffness in patients with type 2 diabetes.

### Sex-dependent association between LBP and arterial stiffness

This study further revealed that the effect of serum LBP levels on aortic PWV is modified by sex and that there is a significant association between serum LBP levels and aortic PWV in men only. A number of studies have shown sex-dependent association of arterial stiffness with metabolic risk factors, such as visceral adiposity [18], the metabolic syndrome, and its components [47, 48]. In previous studies on the LPS-related factors, sex-adjusted association was observed between LBP/soluble CD14 and aortic stiffness [41], carotid intima-media thickness [15], and cardiovascular disease [14]. However, unlike the present study, these previous studies did not stratify the data by sex. Several lines of evidence indicate the sex-associated difference in inflammatory responses to LPS-stimulation in neutrophils in vitro models [49, 50] and in mice in vivo models [51], with increased responses in males than in females. Recent studies have also indicated the sex-associated differences in the gut microbiome, one of the major sources of circulating LPS [42], and the sex-dependent effects of diet on the gut microbiota in mice and humans [52]. In a study performed in rats, systemic proinflammatory cytokine levels in response to an oligofructose-supplemented diet were higher in males than in females [53]. Based on these reports, we can hypothesize that men with type 2 diabetes are more prone to the immune response elicited by LPS from the gut microbiota than are women, leading to increased arterial stiffness in men. To our knowledge, this study is the first to elucidate the sex-related differences in the association between LBP and arterial stiffness.

### Limitations

This study has several limitations. First, we evaluated the endotoxin-induced inflammation by measuring serum LBP levels, but not by a direct measurement of LPS. However, serum LBP levels, which have been proposed as a surrogate marker of LPS-induced immune response in humans, can be simply and stably measured by enzyme-linked immunosorbent assay [8, 10, 11]. We also did not include the evaluation of the LPS-related factors other than LBP, such as interleukin-6, soluble CD14, and tumor-necrosis factor-α, that could strengthen our study. Second, since this was a cross-sectional study, the causal relationship between LBP and arterial stiffness needs to be confirmed by longitudinal and/or interventional studies. Third, the subjects were receiving statins and/or RAS inhibitors, which could have influenced inflammation, vascular function, and related risk factors. To minimize the impact of these drugs, the presence of these treatments was adjusted for in the multiple regression analysis. Fourth, the sample size was too small in the sex-stratified analysis to conclude with statistical significance the relationship between serum LBP levels and aortic PWV in men after adjusting for log [HOMA-R]. Finally, because our subjects were hospitalized in a university hospital and had inadequate glycemic control, the present results may not be generalized to the entire population of patients with type 2 diabetes.

## Conclusions

This study clearly demonstrated that serum LBP levels are independently and positively associated with arterial stiffness in patients with type 2 diabetes. Our data indicate a close link between LPS/LBP-induced innate immunity and arterial stiffness in these patients. This study further shows that LBP preferentially affects arterial stiffness in men over women, indicating sex-related differences in the link between LBP and arterial stiffness. Further interventional studies are warranted to clarify whether the reduction of serum LPS levels by antibiotics or probiotics would reduce arterial stiffness and the risk of cardiovascular diseases in patients with type 2 diabetes.

## Abbreviations

LPS: lipopolysaccharide
LBP: lipopolysaccharide-binding protein
PWV: pulse wave velocity
BMI: body mass index
CD: cluster of differentiation
TLR: toll-like receptor
hs-CRP: high-sensitivity C-reactive protein
eGFR: estimated glomerular filtration rate
HOMA-R: homeostasis model assessment of insulin resistance
SD: standard deviation
HbA1c: glycated hemoglobin A1c
HDL: high-density lipoprotein
LDL: low-density lipoprotein
RAS: renin-angiotensin system
